# Foreign Correspondence

**Published:** 1868-02-01

**Authors:** 


### *FOREIGN CORRESPONDENCE.*

---

PARIS, *January 2d*, 1868.

*Editor Chicago Medical Journal:*

IT is well known that the conditions favorable for healing of wounds by adhesion and union by the first intention are, clean surfaces, unimpaired vitality, entire cessation of bleeding, perfect coaptation, exclusion of air, light dressing, and only a very slight grade of inflammatory process. One of these conditions, *exclusion of air*, is attracting at present the attention of the medical profession in this country. Dr. Guérin has communicated on this subject to the Academy of Sciences a most interesting paper, in which he advocates the use of an instrument he has invented with the object of obtaining the pneumatic occlusion of the parts undergoing the process of healing. By means of this instrument, open wounds are placed in the same conditions as sub-cutaneous wounds. The apparatus is so constructed as to effect the double purpose of excluding the air, and absorbing the secretions from the parts under treatment. The importance of this method may be judged by the admirable results attained. Pneumatic occlusion has heretofore been applied to four kinds of open wounds. In the first category are comprised tegumentary wounds, and simple surgical operations—that is to say, those which go no deeper than the skin, and the cellular tissue — incisions, section of cicatritial bands, excisions of sub-cutaneous tumors, and extractions of extraneous bodies from articulations. Among the cases mentioned by the author, I will notice that of the extraction of hydatiform concretions of the wrist. By ordinary means, this operation is generally considered as one of the most dangerous in surgery. The patient, a wine-dealer at Vesinet, had been, at several times, under the care of Drs. Velpeau, Nelaton, and Langier. Dr. Guérin performed the operation in presence of several surgeons. On the fourth day the wound was completely healed. The tumor having reappeared six weeks after, owing to several granulations which had been overlooked, the operation had to be renewed. Four days after, the new wound was entirely healed. No accident, nor any symptoms of suppurative inflammation were observed.

To the second category belong grave operations, such as amputation of limbs. One of these operations, performed by Dr. Maisonneuve on a patient who died of cholera a few days after, has showed, upon dissection of the stump, a singular result, and an additional advantage produced by pneumatic occlusion. When the operation was being gone through, Dr. Guérin requested the operator not to tie the arteries, but simply to bend them together with the stump, he having found, by experimenting on animals, that the obliterations of the arteries could be produced by adopting that method. On the fifteenth day subsequently to the operation, the patient having died of cholera, the autopsy revealed the fact, that both the radial and cubital arteries were entirely obliterated. The anxiety felt by Dr. Guérin to get rid of all kinds of extraneous bodies, including ligatures, from wounds submitted to pneumatic occlusion, had led him to employ this method that seems destined to do away with the ligature, which constitutes, in all operations, a most serious complication.

In the third category, we find contused wounds with abrasion of the skin, complicated fractures, and simple ones—that is to say, accompanied with perforation of the integument, the bones simply broken. Our author mentions the case, amongst others, of a child suffering from a complicated fracture of the fore-arm, the fragments of the radius protruding through an opening in the integument. On the third day the cutaneous wound was entirely closed, the consolidation of the fracture took place just as in a simple fracture.

The last category comprises wounds by firearms, with laceration and destruction of the tissues, comminuted fractures and crushing of the bones, wounds which present the gravest complications of traumatic lesions. "On the 28th of August, 1865," says the doctor, "I was called at Reims, by a telegraphic despatch, to see a merchant who was suffering from the laceration of the palm of one of his hands, caused by the explosion of a cartridge. The shot, penetrating the hand, had bruised the flesh, cut the arteries, lacerated the nerves and tendons, and produced a comminuted fracture of the bones. The tattered and shrunken skin gave a full view of the articulations, and the hand, as a whole, presented but a mass without shape, in which the swollen and torn fingers were hardly distinguished. After the first care given by the professors of the medical school at Reims, the ligature of the arteries having been performed, the wound cleansed, and fifteen sutures done, the hand, covered with a suitable bandage, was introduced in the apparatus, which was then placed in connection with the pneumatic receiver at sixty-five degrees. No sooner had the operation, which lasted three hours, come to an end, than the patient fell asleep, and rested till seven o'clock the next morning. On awaking, he was calm, had not felt at all feverish, his hand was but moderately sensitive. Pneumatic occlusion was still carried on by Drs. Gallier and Stappart. I saw the patient eight days after the accident; he had suffered no feverish attack, no traumatic accident whatever, the dead portions of the flesh, the excreted liquids had passed through the pneumatic receiver, and granulations were springing up. On the fourth week, the wound was filled up, and on a level with the surface of the hand. The thirty-fifth day it was completely cicatricized, presenting no traces but the lines marking the points of the union between the parts. Five months after, the healed man was presented to the Academy of Medicine, and every one there acknowledged that the hand looked like a natural one, the cicatrix offered the appearances of natural skin."

These facts suffice to place in evidence the physiological properties, and the practical advantages of pneumatic occlusion applied to the treatment of open wounds. In a few words, in the most normal conditions this method produces cicatrization without traumatic or symptomatic fever, without any suppurative inflammation, brings about immediate re-organization. In less favorable conditions, as when the wound has been open for some time, or when it contains extraneous substances, or when, again, it is complicated with former morbid states, pneumatic occlusion can not prevent a certain degree of suppurative inflammation, but continued aspiration effectually arrests all accidents resulting from the putrefaction and resorption of the altered liquids. At all events, the method proposed by Dr. Guérin favors and renders more rapid the cicatrization or consecutive organization of the wounds.

Practitioners are well aware that *Cod-liver Oil*, which has now become an important element in Materia Medica, unfortunately possesses a taste, the repugnance to which many patients can not overcome. Dr. Roland has endeavored, and not, it seems, without success, to remove this obstacle by riding the oil of its characteristic taste. His receipt is as follows: *Cod-liver Oil*, 100 grammes (21 drachms); *Alcohol*, at forty degrees of Baume's areometer, 60 grammes (12½ drachms); *Essence of Peppermint*, 3 grammes (45 grains). By mixing these ingredients, an emulsion is formed, which is administered at the dose of three table-spoonsfull a-day. The proportion of these ingredients may be varied according to the taste of the patient. Dr. Roland states that he has obtained very satisfactory results from this mixture.

W.

[We find (*Reporter*, January 11) since the receipt of the foregoing letter, a description of two forms of apparatus suggested for the occlusion of wounds from the air, in the manner described by our correspondent.—Ed.]

M. Guérin, and M. Maisonneuve, have each submitted to the Academy of Sciences an instrument for the protection of wounds from the action of air. The object proposed by M. Guérin, is to reduce exposed wounds to the condition of subcutaneous wounds, so as to obtain union by first intention. The apparatus consists in a hemispheric glass balloon, with three tubulated orifices. One of these communicates with a manometer, which indicates the degree of vacuum established in the globe. The second communicates with the patient; the third with a central reservoir of vacuum, to which all the individual apparatus in a ward may be attached.

The manometer consists of a barometric tube, and a glass globe communicating with it, and both contain mercury. The globe is plunged in the hemisphere before-mentioned. When the air is exhausted from the hemisphere, the mercury falls in the tube and the globe. By means of this apparatus the wound is in contact with a vacuum instead of air, and at the same time aspiration is continually exerted on the fluids exuding on it surface.

M. Maisonneuve's apparatus is more especially designed to meet this latter indication. An india-rubber cap, furnished with a tube, is placed over a wound (especially a stump after amputation). The tube is in communication with a flask, that, by means of another tube, is connected with an air-pump. This exhausts at once the flask and the cap, and the latter collapses, and, pressed by the air against the surface of the wound, affords it the most complete protection. At the same time the fluids are incessantly drawn off into the flask.

